# Long-Term Treatment Outcomes of PEERS^®^ for Preschoolers: A Parent-Mediated Social Skills Training Program for Children with Autism Spectrum Disorder

**DOI:** 10.1007/s10803-021-05147-w

**Published:** 2021-07-24

**Authors:** Isita Tripathi, Jasper A. Estabillo, Christine T. Moody, Elizabeth A. Laugeson

**Affiliations:** 1 Semel Institute for Neuroscience and Human Behavior, University of California, 300 UCLA Medical Plaza, Los Angeles, CA 90095-6967 USA; 2 Harvard Medical School, 25 Shattuck St, Boston, MA 02115 USA; 3 Department of Psychiatry and Biobehavioral Sciences, University of California, 300 UCLA Medical Plaza, Los Angeles, CA 90095-6967 USA; 4 Department of Psychology, University of California, 1264 Franz Hall, Los Angeles, CA 90095-1759 USA

**Keywords:** Social skills, Autism, PEERS^®^, Long-term outcomes, Preschool

## Abstract

Although parent-assisted social skills interventions may reduce early social challenges in preschool-aged children with autism spectrum disorder (ASD), limited research has explored whether intervention gains maintain several years after treatment. This study examined the durability of PEERS^®^ for Preschoolers, a parent-mediated social skills training program for preschool-aged children with ASD and other social challenges. Twenty-nine parents reported on child and family outcomes 1–5 years following treatment. Results demonstrated maintenance of treatment gains on measures of ASD-related social impairments including social communication, social responsiveness, social motivation, and peer engagement. Post-treatment improvements in problem behaviors and parenting stress were not maintained at long-term follow-up. Implications of these results are discussed.

Individuals with autism spectrum disorder (ASD) are known to demonstrate pervasive impairments in social communication that often emerge in early childhood and persist throughout the lifespan (American Psychiatric Association [APA], 2013; Carter et al., 2005). Young children with ASD may display limited shared and imaginative play, lower frequency of social initiation with peers, and reduced emotional responsiveness toward playmates (APA, 2013). Through the early school years, social isolation becomes increasingly common as children with ASD struggle to effectively engage with their peers (Kasari et al., 2011; Rotheram-Fuller et al., 2010) or learn developmentally appropriate social behavior through naturalistic interactions (Chevallier et al., 2012). Alongside difficulty with social communication, children with ASD often exhibit challenging behaviors, repetitive language or motor stereotypes, and specialized, fixated interests that further interfere with peer interactions (APA, 2013).

These symptoms and challenges in social communication can have significant negative impacts on children and their families. Children with ASD tend to experience limited peer relationships and poorer friendship quality compared to typically developing peers (Kasari et al., 2011; Koegel et al., 2012). Delays in social relatedness among children with ASD have also been linked to strained family functioning, including overall parenting stress and parent–child relational issues (Davis & Carter, 2008; Schieve et al., 2007). Many parents cite their child’s social communication impairments as one of their greatest sources of stress during developmental years (Hayes & Watson, 2013; Schieve et al., 2007).

Further, persistent difficulties with social interaction and elevated parenting stress may result in long-term negative impacts on child development. Among youth with ASD, social difficulties have been associated with poor mental health, educational, and vocational outcomes through childhood, adolescence, and young adulthood (Ashbaugh et al., 2017; Chen et al., 2015; Rodriguez et al., 2020). Among parents, evidence suggests that elevated parenting stress adversely impacts child functioning and parenting behaviors (Neece et al., 2012; Hutchison et al., 2016). These challenges experienced by youth with ASD and their families highlight the need to intervene early to address social communication difficulties, and consequently improve trajectories across the lifespan. Many parents have indicated a desire for more psychoeducation, social support, and practical strategies to facilitate their children’s social engagement (Schieve et al., 2007; Whitaker, 2002). Thus, interventions that target both early peer relationships and complementary parent strategies are needed.

Early social skills interventions may provide an optimal platform to address the needs of children with ASD while equipping parents with appropriate psychoeducation and skills. However, meta-analyses suggest that interventions addressing social impairments in the preschool population (i.e., children aged 3–5 years) are extremely limited and most have not undergone rigorous testing (Reichow & Volkmar, 2010; Wolstencroft et al., 2018). Those few studies that have utilized open or randomized control trials reinforce findings of single-subject and quasi-experimental studies, underscoring the potential of social skills interventions to make meaningful advancements in the social behaviors of young children with ASD (Park et al., in press; Ichikawa et al., 2013). While improvements in social initiation, emotional regulation, and reciprocal play have been found, small sample sizes across many studies in the current evidence base have limited more comprehensive analyses of treatment outcomes (Reichow & Volkmar, 2010; Wolstencroft et al., 2018).

Additionally, while research suggests that responsive parenting practices may promote social engagement in young children with ASD (Caplan et al., 2019; Ruble et al., 2008), reviews indicate that few early childhood social skills programs include parent involvement (Rao et al., 2008; Reichow & Volkmar, 2010). Parent involvement in social skills treatment often includes learning about socialization and development, obtaining effective strategies to direct and supervise social interactions, and practicing social coaching within the clinical setting (Frankel et al., 2010; Laugeson et al., 2012). Parent involvement in social skills programs has been associated with more robust and durable treatment gains across developmental stages, as well as continued opportunities for growth and generalization outside the intervention (DeRosier et al., 2011; Frankel et al., 2010; Laugeson et al., 2012; Reichow & Volkmar, 2010). These gains are largely attributed to increased parental support for the child in developing peer networks and practicing skills outside of the clinical setting (Laugeson et al., 2012; Wolstencroft et al., 2018).

In early years, results from parent-mediated social communication interventions have shown improvements in early socialization skills among preschool-aged children (Aldred et al., 2004; Ingersoll & Gergans, 2007; Schertz & Odom, 2006). However, these social communication interventions represent a fundamentally different treatment approach than traditional social skills training and may be better encompassed by the label of naturalistic developmental behavioral interventions (Schreibman et al., 2015). Such programs rely on naturalistic teaching opportunities during dyadic play interactions or everyday routines to model and reinforce desired social communicative behaviors, including not only speech and gestures, but also joint attention, joint engagement, social reciprocity, turn-taking, and imitation, in an effort to promote a pathway toward normalized social learning in young children with ASD (Schreibman et al., 2015). Programs utilizing a traditional social skills training framework often rely on more direct, didactic-style lessons to discuss, demonstrate, and practice discrete and targeted social skills (Gunning et al., 2019; Wolstencroft et al., 2018). As such, there is also a greater focus on concrete rehearsal of pragmatic language and social behavior that children may later use in specific social contexts (e.g., greeting a friend, asking for a turn), in addition to supported teaching of more advanced concepts, such as perspective-taking and social norms, rules, or cues (Krasny et al., 2003; Rao et al., 2008; Reichow & Volkmar, 2010). Within preschool-age social skills interventions, only two studies have integrated parent-training through a concurrent parent group that runs simultaneously with the child group (Ichikawa et al., 2013; Park et al., in press). Results in both studies suggested benefits, although both programs were limited by small sample sizes and lack of comparison groups.

Despite promising preliminary research that social skills programs produce positive benefits, meta-analyses reveal that existing models often neglect elements of effective social skills interventions for individuals with ASD across the lifespan (Gates et al., 2017; Wolstencroft et al., 2018). A recent review describing social skills programs in children, adolescents, and young adults with ASD indicates that many of the most effective programs include instruction based on concrete steps and rules, behavioral modeling and rehearsals, performance feedback, and homework assignments to promote generalization (Moody & Laugeson, 2020). These elements have been denoted as important for acquisition of skills and generalization in preschool-age programs as well, across both literature reviews and evidence-based clinical trials (Gunning et al., 2019; Ichikawa et al., 2013; Park et al., in press). The Program for the Education and Enrichment of Relational Skills (PEERS^®^) for Preschoolers integrates each of these recommended elements into a parent-mediated social skills intervention program. An initial clinical trial of PEERS^®^ for Preschoolers indicated significant improvements in overall social skills and significant decreases in overall problem behaviors on age- and gender-normed measures. Social responsiveness and social impairments specific to autism were also found to significantly improve over the course of the intervention. Analysis of certain subdomains indicated improvements in social cognition, social communication, and social motivation. Children were also attending a greater number of playdates per month, suggesting a potential increase in social engagement (Park et al., in press).

Very limited research has also investigated whether young children maintain treatment gains beyond completion of social skills intervention (Gates et al., 2017; Gunning et al., 2019; Reichow & Volkmar, 2010). Given changes in social demands during childhood, maintenance of skills is an important outcome of early social skills programs to determine whether families experience continued benefit (Mandelberg et al., 2014; Rao et al., 2008). A small number of single-subject preschool programs have evaluated outcomes 1–6 months after completion (Gena et al., 2005; Laushey & Heflin, 2000; Nikopoulos & Keenan, 2007) and found evidence of maintenance in this brief follow-up period. Notably, none of the interventions tracking maintenance were parent-mediated or parent-assisted.

Although research has not robustly established long-term outcomes in preschool-aged interventions, follow-up studies on parent-assisted programs for other age groups indicate promising outcomes. A 6-year follow-up study of a parent-assisted early intervention program focusing on social communication among 2–4 year old children noted decreases in symptom severity during both intervention and follow-up periods (Pickles et al., 2016). Furthermore, long-term follow-up of the UCLA PEERS^®^ for Adolescents program similarly demonstrated improvement in social functioning pre- to post-intervention with maintenance 1–5 years later on all outcome measures (Mandelberg et al., 2014). Despite evidence of longitudinal improvements in early intervention and school-aged programs, there is limited understanding about the durability of social skills interventions for preschool-aged children with ASD, particularly for programs that include an active parent training component.

To address the scarcity of relevant research in this area, the present study sought to examine child and associated parent outcomes 1–5 years after completing the PEERS^®^ for Preschoolers program. Based on the impact of parent involvement on child outcomes (DeRosier et al., 2011; Frankel et al., 2010) and research on long-term outcomes following the PEERS^®^ for Adolescents program (Mandelberg et al., 2014), maintenance of treatment gains was expected on measures of child and parent functioning related to social skills and play interactions.

## Methods

### Participants

Based on evidence of the improved social skills outcomes following PEERS^®^ for Preschoolers (Park et al., in press), the program was expanded and offered clinically for children 4–6 years of age and their parents. Participants in the present study were recruited from a database of families in previous PEERS^®^ for Preschoolers clinical groups who had agreed to be contacted for enrollment in future research. Original criteria for participation in PEERS^®^ for Preschoolers required that the child had (a) difficulty with peer interactions, (b) adequate expressive language, (c) no physical or medical conditions that would interfere with treatment, and (d) a parent fluent in English who was willing to attend the program. Participant eligibility for enrollment was assessed using a phone screen and confirmed through an in-person intake conducted by a postdoctoral psychology fellow or licensed clinical psychologist. Adequate expressive language was determined based on clinical judgement during intake to ensure that participants could meaningfully participate and benefit from group activities involving verbal skills. Children were required to demonstrate comprehension and expression of sentence-level speech, with spontaneous expressive use of 4-word phrases including verbs. This was assessed through interactive play activities, including ability to respond to developmentally appropriate instructions and questions within natural interaction.

Parents completed assessment measures at intake (T1) and directly after completion of the intervention (T2). Criteria for participation in the current study required successful completion of pre- and post-intervention measures between 1 and 5 years prior to study recruitment (i.e., between October 2015 and February 2019). The current sample was limited to children with a previous ASD diagnosis. Of the 45 eligible participants, 64.4% (*n* = 29) completed follow-up measures between November 2019 and March 2020. Demographic information for study participants (“Completers”) and eligible participants who did not complete follow-up (“Non-Completers”) is presented in Table 1. A priori analyses indicated no group differences between Completers and Non-Completers on any child or parent variables (See Table 1). Independent samples *t*-tests further indicated no significant differences in program attendance, T1 scores, T2 scores, or change in scores between T1 and T2 between these groups (*p* > 0.05). Although ASD diagnosis was not independently confirmed by the study team, 93% of study participants (*n* = 27) had clinically significant autistic behaviors (*T* ≥ 60) on the Social Responsiveness Scale, Second Edition (SRS-2; Constantino & Gruber, 2012) at baseline, and all participants had been given a previous diagnosis of ASD by a reliable medical or mental health professional (e.g., psychologist, developmental pediatrician, Regional Center), further supporting the presence of ASD symptomatology.

**Table 1:** Demographic characteristics of follow-up assessment completers versus non-completers

Variable	Completers (*n* = 29)			Non-completers (*n* = 16)		
	*M*	*SD*	%	*M*	*SD*	%
Age						
Baseline (T1)	4.99	0.85		4.81	0.50	
Post-treatment (T2)	5.46	0.82		5.24	0.53	
Follow-up (T3)	7.88	1.39		–	–	
Gender						
Males			79			75
Females			21			25
Child ethnicity						
Caucasian			35			25
Latinx/Hispanic			14			13
African American/Black			0			6
Asian			21			19
Native American			3			0
Middle Eastern			3			12
Multi-racial			17			19
Other			7			6
Diagnoses						
ASD			100			100
Anxiety			3			0
ADHD			24			6
Other			7			12
Parent relationship to child						
Mother			86			81
Father			14			19
Parent highest education						
Some college			7			0
Bachelor’s degree			38			56
Advanced graduate degree			55			38

“Other” diagnoses include sensory processing disorder, non-verbal learning disorder (visual motor processing), tic disorder, and apraxia

*M* mean, *SD* standard deviation

Parents completed follow-up measures between 16 and 56 months post-intervention (*M* = 34.73, *SD* = 11.28). Families who participated in the current follow-up study displayed consistent attendance during the original PEERS^®^ for Preschoolers program, attending on average 92% of the 16-sessions (*M* = 14.76, *SD* = 1.30). At the start of the intervention, the majority of children (*n* = 25, 86%) fell in the preschool age range (i.e., 3–5 years old). Four additional children (14%) started at age 6; these children were clinically determined as appropriate for the PEERS^®^ for Preschoolers program based on their developmental stage and/or social contexts. At follow-up, the average age of participants was 7.88 years (*SD* = 1.39, range: 5–11).

### Procedure

Participants who were deemed eligible to participate in the current follow-up study were contacted via phone or e-mail. Informed consent was obtained through a secure online survey platform (UCLA Qualtrics). Parents were then administered a battery of psychosocial measures via UCLA Qualtrics. A $25 gift card incentive was disbursed to all participants for successful completion of forms. All procedures in the study were approved by and performed in compliance with the ethical standards of the UCLA Institutional Review Board.

#### Intervention

PEERS^®^ for Preschoolers is a parent-mediated social skills intervention for young children with ASD and other social challenges that teaches play and friendship-making skills. To address the common social impairments and developmental needs of young children with ASD, the program adapts key features of the evidence-based PEERS^®^ curricula for adolescents and young adults (Laugeson, 2017; Laugeson et al., 2012), such as didactic lessons with concrete rules and steps of ecologically valid social skills, role-play demonstrations, behavioral rehearsal exercises, and parent-supported homework assignments and reviewal. An overview of the PEERS^®^ for Preschoolers curriculum can be found in Table 2. Groups were led by licensed clinical psychologists and postdoctoral fellows with support from a team of behavioral coaches, consisting of clinical psychology doctoral students and undergraduate research assistants who had experience working with youth with ASD. Coaches were trained and supervised by clinical staff to monitor treatment fidelity, assist with didactic instruction during role-play demonstrations, and provide in-vivo performance feedback to children and parents during behavioral rehearsal activities.

**Table 2:** Overview of the PEERS^®^ for Preschoolers curriculum

Session	Concept	Child didactic lesson	Parent didactic lesson
1.	Communication	Program orientation; listening and following directions	Program orientation; characteristics of good friendships; identifying play groups; praising strategies; social coaching tips for listening and following directions
2.	Communication	Meeting and greeting friends	Characteristics of good play groups; creating practice opportunities; social coaching tips for meeting and greeting friends
3.	Turn-taking	Sharing and giving a turn	Assessing quality of play groups; prompting strategies; social coaching tips for sharing and giving a turn
4.	Turn-taking	Asking for a turn	Parent’s role before attending play groups; strategies to provide corrective feedback; social coaching tips for asking for a turn
5.	Sportsmanship	Keeping cool	Preparing for playdates; coaching during playdates; social coaching tips for keeping cool
6.	Sportsmanship	Being a good sport	Suggesting playdates to other parents; social coaching tips for being a good sport
7.	Reciprocal interaction	Show and tell during play	Preparing for playdates; social coaching during playdates; praise and reinforcement strategies; social coaching tips for showing and telling
8.	Play initiation	Asking a friend to play	Social coaching strategies during playdates; social coaching tips for asking a friend to play
9.	Play initiation	Joining a game	Social coaching tips for joining a game
10.	Self-advocacy	Asking to play something different	Social coaching tips for playing something different
11.	Self-advocacy	Asking and giving help	Social coaching tips for asking and giving help
12.	Social pragmatics	Staying in your own space	Social coaching tips for staying in your own space
13.	Social pragmatics	Using an inside voice	Social coaching tips for using an inside voice
14.	Social pragmatics	Using polite words	Social coaching tips for using polite words
15.	Review	Lesson review part I	Review of skills 1–8; review social coaching strategies
16.	Review and graduation	Lesson review part II; graduation	Review of skills 9–14; providing social coaching

This table has been adapted from the PEERS^®^ for Preschoolers clinical trial (Park et al., in press)

Parents and children met in separate simultaneous groups of approximately 8–10 participants for 90-minute sessions over the course of 16 weeks. Each session focused on a specific, concrete social behavior, with lessons organized in a stepwise fashion to ensure continued practice and build upon previously taught skills. Didactic content in the child group was delivered through a puppet show to model the targeted social skill and maximize child engagement. During the didactic, children took turns practicing the skill in front of peers. Afterwards, children practiced skills in the context of familiar group-based games (e.g., “Musical Chairs,” “Red Rover”) while receiving coaching and feedback from the treatment team. In the parent group, the group leader discussed homework completion, provided individualized feedback based on parent needs, and reviewed the targeted skill for each session. Parents received broad psychoeducation on prosocial and challenging behaviors, socialization practices, and friendship development, with a focus on becoming effective social coaches for their children. During the final 20 minutes of each session, parents and children were reunified for mock one-on-one playdates with other participants. These interactions allowed parents to practice social coaching strategies with the support of the treatment team, while children practiced using targeted skills during play. Homework was assigned each week, which included completing playdates and practicing skills with feedback from the parents. Participants were instructed to have playdates with peers outside of the treatment group, in lieu of in-group playdates, to maintain a positive group dynamic and generalize skills to external social contexts. Members of the treatment team monitored treatment fidelity, which was supported through the use of a manual detailing session content, including didactic lesson material, puppet show interaction scripts, and adaptations to familiar games for skills practice.

#### Measures

##### Social Responsiveness Scale, Second Edition: School Age (SRS-2; Constantino & Gruber, 2012)

The SRS-2 is a 65-item informant-rated measure of the presence and severity of ASD-related social impairments in individuals ages 2.5 years through adulthood. The School Age form for children ages 4 to 18 years was used. Parents rated their child’s behaviors using a 4-point scale from 1 (“*Not True*”) to 4 (“*Always True*”)*.* The measure produces a standardized Total Score as well as five subscale scores: Social Communication, Social Cognition, Social Awareness, Social Motivation, and Restricted Interests and Repetitive Behavior (RRB). *T*-scores with a mean of 50 and standard deviation of 10 are utilized, with higher scores indicating greater impairment. Scores of 59 and below are categorized in the typical range. Psychometric properties on the SRS-2 are robust with high internal consistency (alpha coefficients approximately 0.95) and inter-rater agreement (coefficients ranging from 0.72 to 0.82).

##### Quality of Play Questionnaire (QPQ; Frankel & Mintz, 2011)

The QPQ is a 26-item measure that assesses the frequency and quality of one-on-one playdates. Parents reported the number of times that their child invited another child to their home (“Hosted” playdates) or visited another child in their home (“Invited” playdates) during the previous month. The QPQ has become increasingly utilized as an outcome measure for social skill interventions in young children with ASD (Frankel et al., 2011). In addition to evaluating child social engagement, the measure reflects changes in parent initiative to arrange playdates. Further, total number of playdates (i.e., a sum of “Hosted” and “Invited” playdates) differentiates well between elementary school-aged children who were clinic-referred for social problems, who have less than 2.5 playdates per month, and community samples, who have greater than 2.5 playdates per month (Frankel & Mintz, 2011). Although there is no similar benchmark for preschoolers, most participants were school-aged at the time of long-term follow-up. As such, this benchmark is discussed, along with its limitations, in the context of the current study’s results below.

##### Social Skills Improvement System Rating Scales (SSiS; Gresham & Elliot, 2008)

The SSiS is a 79-item informant-rated measure that evaluates social skills and challenging behaviors in children between 3 and 18 years of age. Forms are scored based on national norms for age- and gender-specific categories. In the current study, all participant scores at T1 and T2 were normed using the preschool category (i.e., ages 3–5), including the four participating 6-year-olds, given that preschool most closely matched the developmental level and social contexts of these children. Scores at follow-up were normed to the school age (i.e., ages 5–12) category, which matched all participants’ chronological ages, developmental levels, and social contexts at that time. Raw scores were also utilized to determine functional improvements in social skills over time. Parents reported their child’s behaviors as occurring “*Never*”, “*Sometimes*”, “*Often*”, or “*Always*”. Social skills on the measure are divided into seven subscales: Communication, Cooperation, Assertion, Responsibility, Empathy, Engagement, and Self-control. Problem behaviors are measured across five subscales: Internalizing, Externalizing, Bullying, Hyperactivity/Inattention, and Autism Spectrum Disorder. Standard scores and raw scores are available for overall Social Skills and Problem Behaviors, with higher scores indicating better social functioning or more pronounced behavioral challenges, respectively. A standard score of 85–115 falls within the average range (Gresham & Elliot, 2008). The SSiS is among the most common parent-rated outcome measures for group social skills interventions in children with ASD (Wolstencroft et al., 2018). Parent forms demonstrate high internal reliability with alpha coefficients in the mid-to-upper 0.80s for overall scales and 0.70 or greater for subscales.

##### Parenting Stress Inventory, Fourth Edition, Short-Form (PSI-4-SF; Abidin, 2012)

Abbreviated from the full-length Parenting Stress Inventory, Fourth Edition (Abidin, 2012), the PSI-4-SF is a 36-item questionnaire that measures the magnitude of child-related caregiver stress for parents of children 0 to 12 years of age. The measure assesses caregiver stress in three domains: Parental Distress, Parent–Child Dysfunctional Interaction, and Difficult Child. Parents rate each statement using a Likert scale from 1 (“*Strongly Agree*”) to 5 (“*Strongly Disagree*”). *T*-scores are produced based on population data, with higher scores indicating greater parenting stress. Typical parenting stress levels fall between 39 and 57 *T*-scores. The PSI-4-SF is a widely used tool for studying stress in parents of young children with autism and other developmental disabilities (Davis & Carter, 2008; Hayes & Watson, 2013). Psychometric properties are strong, with internal reliability alpha coefficients ranging from 0.80 to 0.87 for each scale (Abidin, 2012) and evidence of convergent validity with other validated measures (Haskett et al., 2006).

### Data Analytic Plan

Forms with limited missing data (i.e., 1–2 questions) were scored in accordance with the protocols described in the scoring manual for each measure. Three participants were excluded from analysis on the QPQ and SSiS Problem Behaviors, given that their forms were missing a significant amount of data at T2. All other participants had complete data at T1 (baseline), T2 (post-treatment), and T3 (follow-up) on all variables of interest.

All statistical analyses were conducted using SPSS 26.0. To examine treatment effects over time, a series of one-way repeated measures analyses of variance (ANOVA) were conducted with total scores on each treatment measure (i.e., SRS-2, QPQ, SSiS, PSI-4-SF) as the dependent variable. If differences were detected in the overall model, pairwise comparisons were examined between each set of time points (i.e., T1 and T2, T2 and T3, and T1 and T3) to detect changes over the course of intervention and follow-up period. Following significant findings in total scores, a series of repeated measures multivariate analyses of variance (MANOVA) were conducted with subscales on each measure as the respective composite dependent variable. Pairwise comparisons were then examined between each set of time points to analyze treatment effects on measure subscales.

The Benjamini–Hochberg procedure was used to determine significance for all initial ANOVAs and MANOVAs (Benjamini & Hochberg, 1995). *P*-values were sorted and ranked in ascending order. Each rank was divided by the total number of tests (i.e., 10) and multiplied by a false discovery rate of 5% to determine the Benjamini–Hochberg critical value. Reported significant findings had *p*-values below the Benjamini–Hochberg critical value. The alpha level in all post-hoc pairwise comparisons was adjusted for multiple comparisons using a Bonferroni correction.

## Results

Overall, results from repeated measures ANOVAs indicated significant improvements on all child outcomes from baseline (T1) to post-intervention (T2). Treatment gains related to ASD-associated impairments in play and friendship-making skills showed strong maintenance at long-term follow-up (T3); however, treatment gains were not sustained on developmentally-normed measures of social skills and problem behaviors. Some significant improvements in parenting stress were observed after treatment; however, scores in the long-term did not significantly differ from those at baseline or post-intervention. See Table 3.

**Table 3:** Summary of baseline (T1), post-treatment (T2), and follow-up (T3) outcomes

Measure	T1		T2		T3		*p*-value
	*M*	*SD*	*M*	*SD*	*M*	*SD*	
SRS Total Score (*n* = 29)	75.45	9.95	66.86**	10.38	68.34‡	11.52	< 0.001
QPQ Total Playdates (*n* = 26)	1.65	1.74	3.35**	2.59	2.73‡	2.22	0.001
SSiS age-normed Social Skills (*n* = 29)	82.14	10.91	90.34**	9.72	80.41§	11.26	< 0.001
SSiS raw Social Skills (*n* = 29)	63.86	14.08	75.45**	13.21	73.59‡	13.24	0.007
SSiS age-normed Problem Behaviors (*n* = 26)	116.69	13.58	108.69**	12.38	119.08§	17.58	0.002
SSiS raw Problem Behaviors (*n* = 26)	34.69	11.77	27.58**	10.45	32.69§	14.40	0.015
PSI Difficult Child (*n* = 29)	62.76	9.23	58.24**	8.74	59.72	8.21	< 0.001

*p*-Values in the last column indicate significant differences in the overall model looking at change across timepoints for each measure

**Significant difference between T1 and T2 value, *p* < 0.05, representing treatment gains over course of treatment

^‡^Significant difference between T1 and T3 value, *p* < 0.05, representing maintenance of treatment gains over follow-up period

^§^Significant difference between T2 and T3 value, *p* < 0.05, representing loss of treatment gains over follow-up period

### Social Responsiveness

**Fig. 1:**
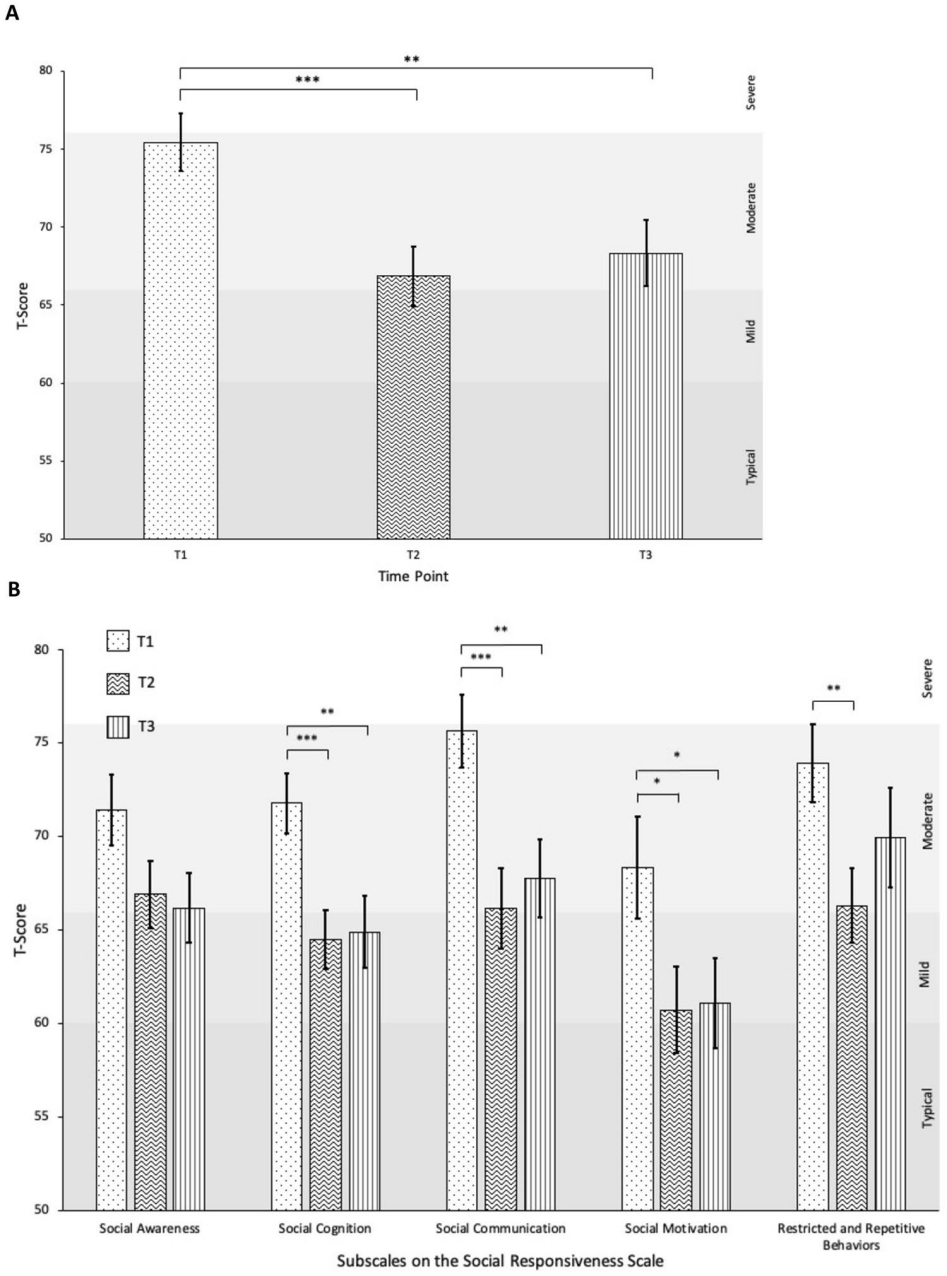
Change in SRS-2 Total Score (**A**) and subscales (**B**) over time. *p < 0.05, **p < 0.01, ***p < 0.001

Significant differences in ASD-related social impairments were found on the SRS-2 across time, *F*(2,56) = 12.39, *p* < 0.001, partial η^2^ = 0.31. Follow-up comparisons showed significant improvements from T1 (*M* = 75.45, *SD* = 9.95) to T2 (*M* = 66.86, *SD* = 10.38), *p* < 0.001. Scores at T3 (*M* = 68.34, *SD* = 11.52) remained significantly different from T1 (*p* = 0.003) with no significant differences from T2 (*p* > 0.05), suggesting that treatment gains maintained over the follow-up period. MANOVA analyses including all SRS-2 subscales also indicated significant changes over time, Pillai’s trace *V* = 0.46, *F*(10, 106) = 3.18, *p* = 0.001, partial η^2^ = 0.23. Post hoc analyses with Bonferroni corrections showed significant improvements in ASD-related social impairments from T1 to T2 on all subscales (*p* < 0.05) except Social Awareness, which showed no change across time points (*p* > 0.05). Maintenance was demonstrated on the Social Cognition, Social Communication, and Social Motivation subscales, as evidenced by no significant differences between T2 to T3 (*p* > 0.05), as well as continued significant differences between T1 and T3 (*p* < 0.05). Scores on the Restricted and Repetitive Behaviors subscale displayed some moderate returns toward baseline levels, such that scores at T3 (*M* = 69.93, *SD* = 14.40) did not significantly differ from either T1 (*M* = 73.90, *SD* = 11.26, *p* > 0.05) or T2 (*M* = 66.31, *SD* = 10.77, *p* > 0.05). See Fig. 1.

### Social Engagement

**Fig. 2:**
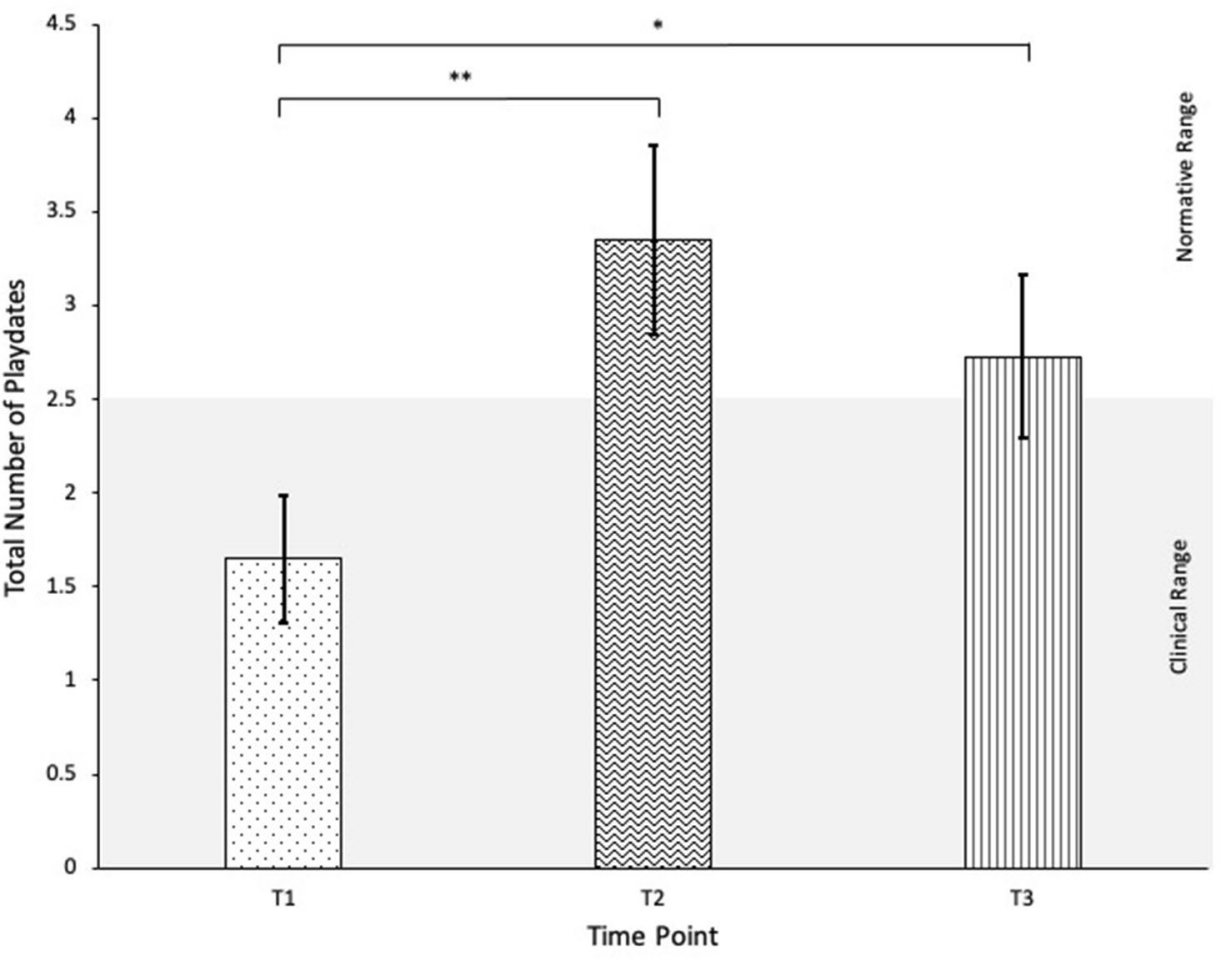
Change in QPQ Total Playdates over time. *p < 0.05, **p < 0.01

The frequency of playdates in the previous month, as measured by a composite sum of Hosted and Invited playdates on the QPQ, exhibited significant changes over time, *F*(2,19) = 7.74, *p* = 0.001, partial η^2^ = 0.24. Post hoc comparisons showed significant increases in the frequency of child playdates per month from T1 (*M* = 1.65, *SD* = 1.74) to T2 (*M* = 3.35, *SD* = 2.59, *p* = 0.001), with playdate frequency at T3 (*M* = 2.73, *SD* = 2.22) remaining significantly different from T1 (*p* = 0.042) and maintaining from T2 (*p* > 0.05). See Fig. 2.

### Social Skills

**Fig. 3:**
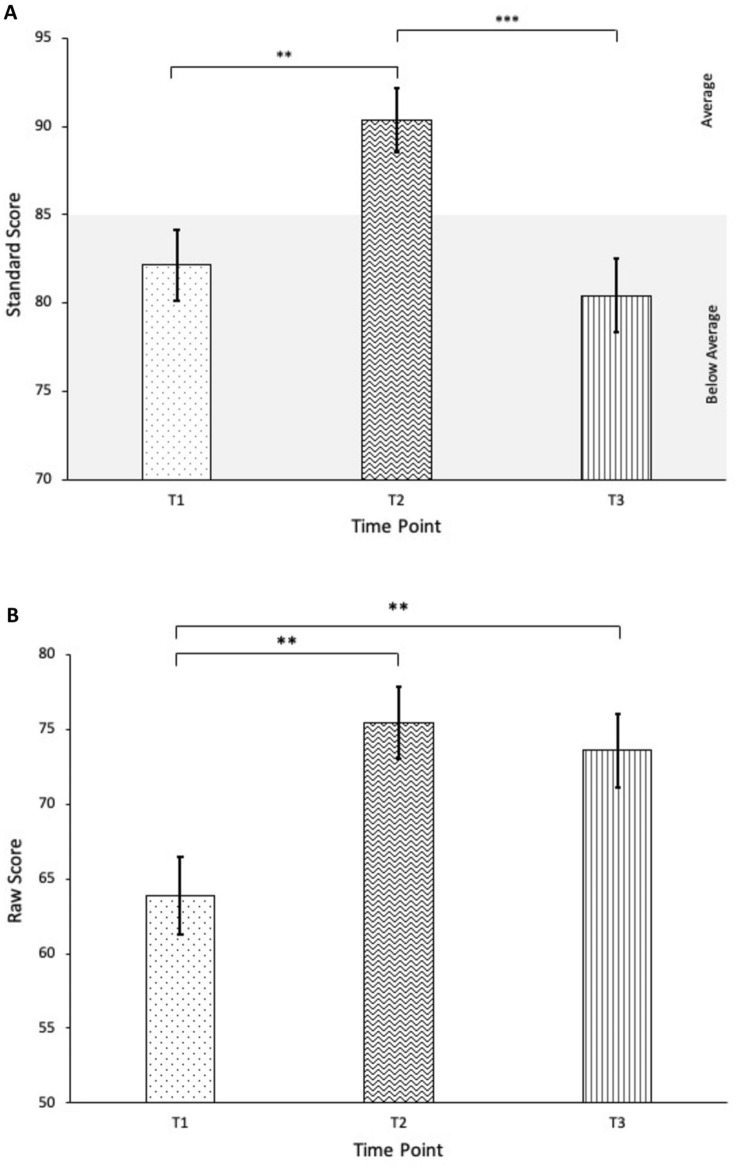
Change in SSiS Social Skills standard scores (**A**) and raw scores (**B**) over time. **p < 0.01, ***p < 0.001

Social Skills on the SSiS also showed changes across time points on both standard scores, *F*(2,27) = 16.58, *p* =  < 0.001, partial η^2^ = 0.55, and raw scores, *F*(2,27) = 5.95, *p* = 0.007, partial η^2^ = 0.31. Although standard scores exhibited improvements from T1 (*M* = 82.14, *SD* = 10.91) to T2 (*M* = 90.34, *SD* = 9.72, *p* = 0.006), treatment gains did not maintain at follow-up (*p* < 0.001), such that scores at T3 (*M* = 80.41, *SD* = 11.26) did not significantly differ from baseline levels (*p* > 0.05). In contrast, significant increases in raw scores from T1 (*M* = 63.86, *SD* = 14.08) to T2 (*M* = 75.45, *SD* = 13.21, *p* = 0.007) were sustained at T3 (*M* = 73.59, *SD* = 13.24); raw scores at T3 did not significantly differ from T2 (*p* > 0.05), yet remained significantly different compared to T1 (*p* = 0.009), indicating maintenance in the long-term. The discrepant results suggest that functional improvements in social skills were sustained over time; however, acquisition of new social skills over the follow-up period did not keep pace with that of an age-matched population of typically developing peers. See Fig. 3.

Further analyses of SSiS subscales revealed changes that approached significance, Pillai’s trace *V* = 0.40, *F* (14,102) = 1.79, *p* = 0.050, partial η^2^ = 0.20. However, findings should be approached with caution, as the *p*-value after Benjamini–Hochberg correction was equal to the critical value. Post hoc analyses with Bonferroni corrections revealed significant improvements on the Cooperation subscale from T1 to T2 (*p* = 0.032) that maintained at T3 (*p* > 0.05 from T2 and *p* = 0.001 from T1). While scores on the Self-control, Engagement, and Responsibility subscales also indicated significant improvements from T1 to T2 (*p* < 0.05), scores at follow-up did not significantly differ from either T1 or T2 (*p* > 0.05), suggesting moderate returns to baseline. No significant differences were found on the Assertion, Empathy, or Communication subscales across time points (*p* > 0.05).

**Fig. 4:**
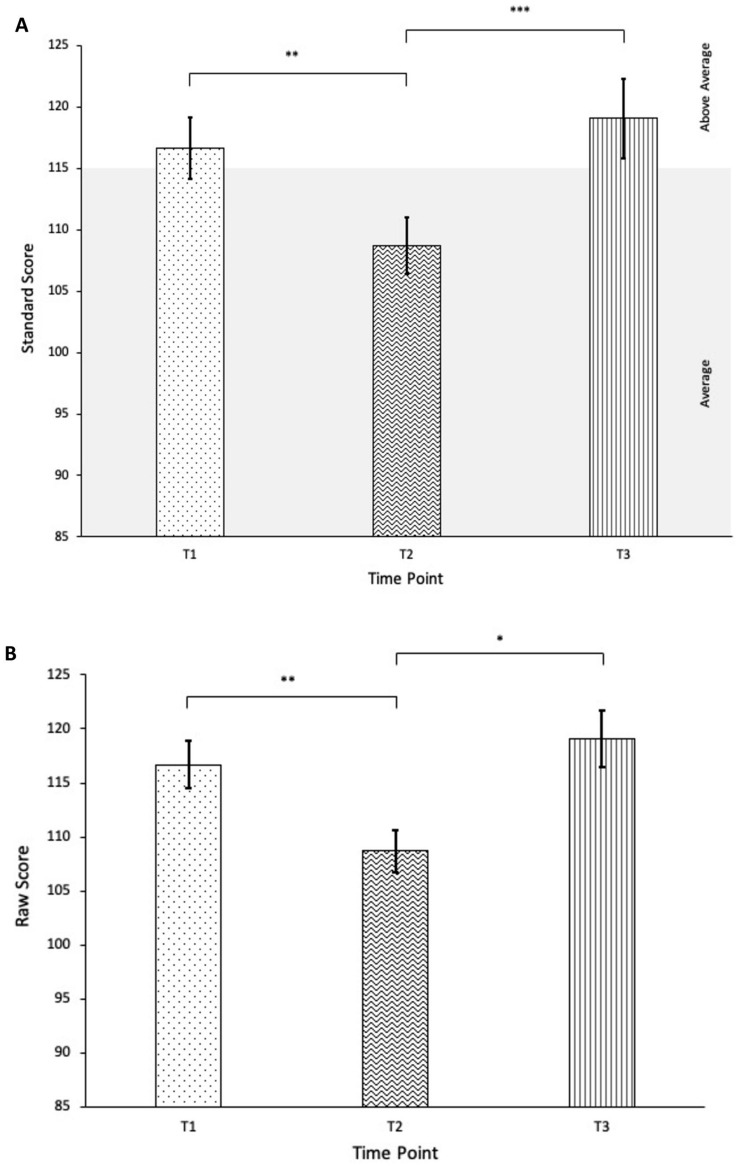
Change in SSiS Problem Behaviors standard scores (**A**) and raw scores (**B**) over time. *p < 0.05, **p < 0.01, ***p < 0.001

Problem Behaviors on the SSiS also displayed significant changes across time points. Mauchly’s test of sphericity was significant; therefore, Greenhouse–Geisser correction was used on both standard scores, *F*(1.54,38.61) = 8.90, *p* = 0.002, partial η^2^ = 0.26, and raw scores, *F*(1.51,37.69) = 5.39,* p* = 0.015, partial η^2^ = 0.18. Post hoc analyses with Bonferroni corrections revealed significant improvements in standard scores from T1 (*M* = 116.69, *SD* = 13.58) to T2 (*M* = 108.69, *SD* = 12.38, *p* = 0.008). However, treatment gains did not maintain from T2 to T3 (*M* = 119.08, *SD* = 17.58, *p* < 0.001), and scores did not significantly differ from baseline levels (*p* > 0.05). A similar pattern was observed with raw scores, as there was a significant reduction in parent-reported problem behaviors from T1 (*M* = 34.69, *SD* = 11.77) to T2 (*M* = 27.58, *SD* = 10.45), *p* = 0.008. Raw scores also exhibited a significant return in problem-behaviors from T2 to T3 (*M* = 32.69, *SD* = 14.40, *p* = 0.016), such that the presence of problem-behaviors at follow-up no longer significantly differed from baseline, *p* > 0.05. See Fig. 4.

Overall, MANOVA analyses including all Problem Behavior SSiS subscales also indicated significant changes over time, Pillai’s trace *V* = 0.52, *F*(10,94) = 3.29, *p* = 0.001, partial η^2^ = 0.26. Similar to overall Problem Behaviors, the Hyperactivity and Inattention subscale showed significant decreases from T1 to T2 (*p* = 0.005), but these improvements did not maintain from T2 to T3 (*p* = 0.035) and scores at T3 did not significantly differ from baseline levels (*p* > 0.05). Scores on the Externalizing and Bullying subscales significantly improved from T1 to T2 (*p* < 0.05) but displayed moderate return to baseline levels in the long-term, such that scores at T3 did not significantly differ from T1 or T2 (*p* > 0.05). No differences between T1 and T2 were found (*p* > 0.05) on the Internalizing subscale; however, scores at T3 were significantly higher than at T2 (*p* = 0.025). Significant decreases from T1 to T2 (*p* < 0.001) on the Autism Spectrum Disorder subscale were found to maintain at T3 (*p* > 0.05 from T2, *p* = 0.023 from T1).

### Parenting Stress

**Fig. 5:**
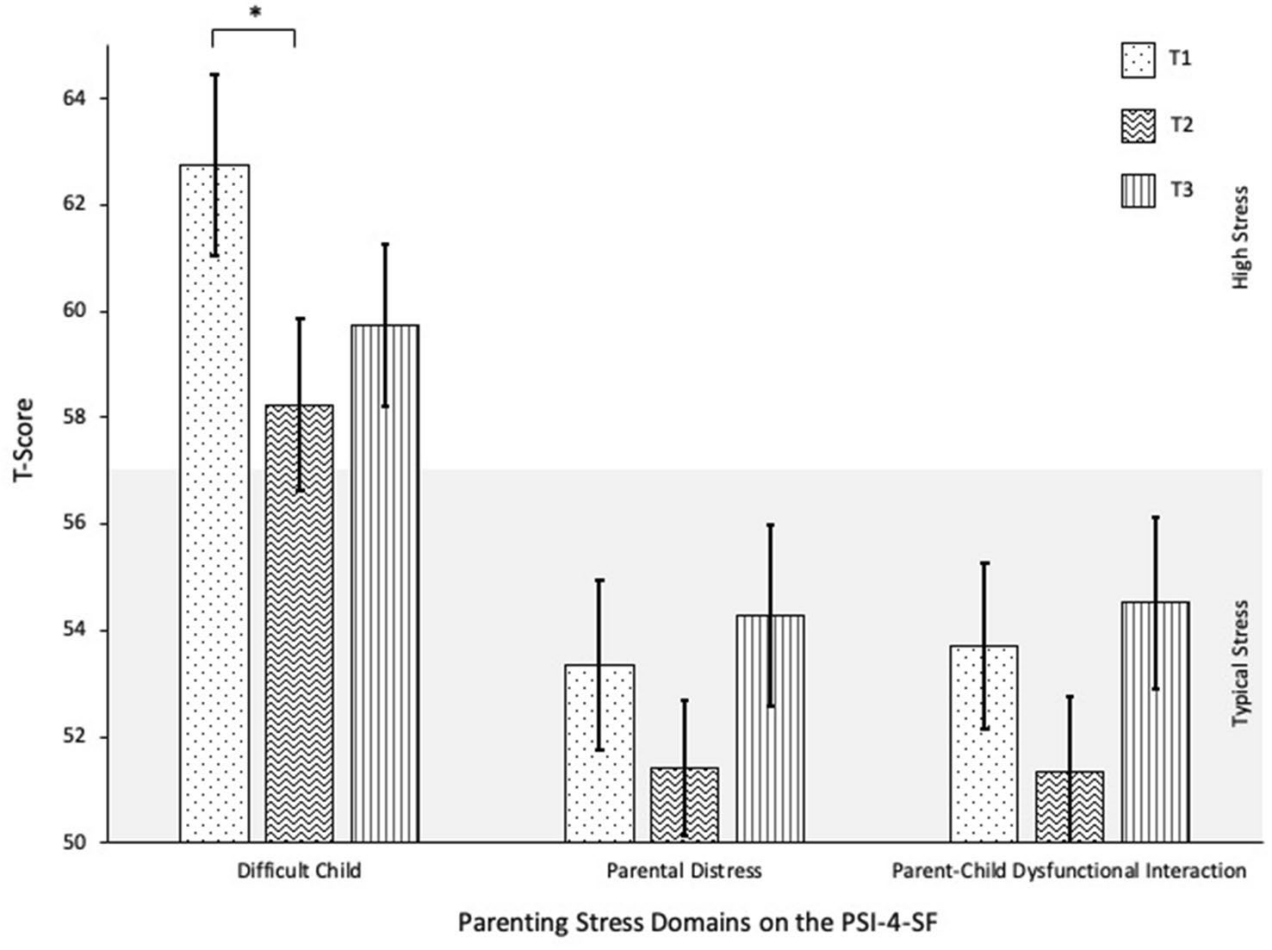
Change in PSI-4-SF domains over time. *p < 0.05

An initial MANOVA including all three parenting stress domains on the PSI-4-SF demonstrated significant differences across time, Pillai’s trace *V* = 0.22, *F*(6,110) = 2.28, *p* = 0.041, partial η^2^ = 0.11. The Difficult Child domain showed significant improvements from T1 (*M* = 62.76, *SD* = 9.23) to T2 (*M* = 58.24, *SD* = 8.74, *p* = 0.037), but scores at T3 (*M* = 59.72, *SD* = 8.21) demonstrated moderate return to baseline levels, with no significant differences from either T1 or T2 (*p* > 0.05). The Parental Distress and Parent–Child Dysfunctional Interaction domains were not found to have any significant differences across time points (*p* > 0.05). See Fig. 5.

## Discussion

Findings from the current study indicate durable, long-term improvements in social behaviors and outcomes of young children with ASD participating in the PEERS^®^ for Preschoolers program. Social skills and ASD-related social impairments in play and friendship-making skills were found to not only improve but maintain 1–5 years after intervention. Treatment gains were less robust over the follow-up period when considering problem behaviors and social skills normed to the broader age-matched population. Findings suggest that long-term maintenance in these areas may require increased or ongoing support adapted to the changing social context and increased social demands during elementary school years. Overall, results from this study are quite encouraging, particularly given the limited research on long-term impacts of evidence-based, parent-mediated social skills interventions for the preschool-aged group.

### Social Outcomes

Social behaviors and functioning were found to improve and maintain over time. Children showed sustained improvement in social responsiveness on the SRS-2, a measure widely used to monitor ASD symptoms over time (Wolstencroft et al., 2018). While scores remained in the Moderate range of severity, the significant improvements post-intervention in ASD-related social impairments were maintained at follow-up. In addition, the total number of playdates that children attended per month also increased and maintained at follow-up, such that participating children were having a comparable amount of playdates to a normative, community-based sample (i.e., > 2.5 playdates per month; Frankel et al., 2010). Since the frequency of a child’s playdates may reflect their number of friends or intensity of friendships (Frankel et al., 2010, 2011), findings suggest a clinically meaningful effect of the PEERS^®^ for Preschoolers program on children’s social engagement in natural contexts outside of the intervention. Moreover, such results have the potential to serve as a pathway for the development of friendships, since early and regular socialization with peers allows children to practice important social communication and play skills (Frankel & Mintz, 2011).

When using standardized scores on the SSiS, maintenance of social skills gains was not observed. However, analysis of raw scores on this measure does indeed indicate that the social skills participants learned were maintained over time. This discrepant set of results suggest that while participants’ social skills behaviors did not regress during the follow-up period, they continued to struggle with mastering new social skills at the same rate as their typically developing peers. Within this context, it is important to note that all children moved from the preschool to school-aged standardization sample at long-term follow-up. Given that social demands shift with social contexts, it would not be expected that PEERS^®^ for Preschoolers would address all of the skills relevant to the new social context of elementary school. Indeed, the shift from preschool to elementary school is significant, representing the movement beyond a primary goal of coordinating play to increased emphasis on conversations, self-regulation, perspective-taking, friendship development, navigating teasing, and establishing broader social acceptance beyond a play-dyad (Denham et al., 2002). As such, individuals with ASD may likely benefit from continued intervention following significant shifts in developmental stage or social context (e.g., from childhood to adolescence, high school to college).

When examining specific domains of social responsiveness and social skills, those related to play-based interactions on both the SRS-2 (i.e., Social Cognition, Social Communication, and Social Motivation) and SSiS (i.e., Cooperation) demonstrated improvement and maintenance across time points. These areas are explicitly targeted in PEERS^®^ for Preschoolers through lessons on turn-taking, self-advocacy, play initiation, and reciprocity within play interactions (See Table 2). It is promising that social skills directly addressed in the curriculum show improvement and maintenance as many as five years post-intervention. Importantly, the teaching strategies utilized in the PEERS^®^ for Preschoolers program require adequate expressive and receptive language skills (i.e., meaningful sentence level speech). Alternative treatment priorities are indicated for children with significant language delays, who may benefit from first engaging in naturalistic-developmental behavioral interventions that promote basic social communication and engagement (Casagrande & Ingersoll, 2017; Dawson et al., 2010).

Long-term maintenance of improvements in social functioning is particularly important for children with ASD due to their established difficulties with generalizing skills across settings and people (APA, 2013). In light of this challenge, many interventions make generalization to outside settings an explicit treatment goal (Frankel et al., 2010; Gena et al., 2005; Laugeson et al., 2012; Mace & Nevin, 2017). Within PEERS^®^ for Preschoolers, parents were trained to use prompting methods and social coaching strategies to incorporate skills into their children’s everyday lives. Sustained improvements for the children who participated in this study may be attributed to this established social coaching framework, which was critical to generalization and continued practice of social skills. Maintenance in the frequency of playdates also highlights the durability of parent involvement in their children’s social worlds, since parents typically arrange playdates and activities for young children (Frankel et al., 2011). Teaching parents to identify play groups, create practice opportunities, and facilitate social interactions may have contributed to continued success in playdates. Consistent with the literature, findings from the present study support high levels of parent involvement in child interventions due to associations with sustained improvements in social skills and peer engagement.

In sum, gains in social functioning were maintained for years following completion of the PEERS^®^ for Preschoolers program. Such findings are encouraging, showing that preschool-aged children with ASD successfully learned and retained meaningful social abilities over time. However, while maintenance was demonstrated, social functioning in the present study did not continue to improve after the termination of the intervention, as has been documented following the PEERS^®^ for Adolescents program (Mandelberg et al., 2014). While social demands continue to increase throughout adolescence and into young adulthood, the fundamental social tasks are rooted in conversational skills throughout both developmental periods, perhaps allowing for continued application of learned skills and subsequent growth following treatment. In contrast, the fundamental social tasks in preschool are based in play, a foundation which gradually shifts to conversation during middle childhood. Alternatively, it is possible that older youth may be better at adapting their previously learned skills to shifting social demands in adulthood, following from some evidence of diminished deficits in cognitive flexibility with age in youth with ASD (van der Bergh et al., 2014). In contrast, younger participants may rely more heavily and concretely on the specific rules and steps included in the curriculum.

### Problem Behaviors and Parenting Stress

In contrast to improvements in social domains, post-treatment improvements were not maintained on measures of problem behaviors, including overall scores on the SSiS Problem Behaviors and the SRS-2 RRB subscale. Decreases in RRBs and problem behaviors after the intervention may have been related to children’s increased ability to demonstrate appropriate communication and play skills, such that appropriate skills acted as an alternative behavior to some challenging behaviors. As discussed above, the changing social demands in the elementary school years may result in a re-emergence of problem behaviors as children with ASD struggle to naturally learn evolving social skills (Denham et al., 2002). Although PEERS^®^ for Preschoolers is a targeted social skills intervention that does not explicitly address challenging behaviors, continued support in this area through other service systems may be helpful to promote broader positive child and family outcomes.

A similar trend was noted on the Difficult Child domain of the PSI-4-SF, which measures parenting stress related to challenging behaviors among children. Scores on the Difficult Child domain have been shown to be correlated with internalizing-externalizing child behaviors and autism severity (Zaidman-Zait et al., 2011), and it is possible that the lack of maintenance in parenting stress outcomes may be related to concurrent increases in child problem behaviors. No significant changes were observed on the other two parenting stress domains, Parental Distress and Parent–Child Dysfunctional Interaction, over the course of treatment or in the follow-up period. However, given that parents rated their stress in the typical range at all three time points on these domains, these null results may not fully represent the potential of significant benefits following PEERS^®^ for Preschoolers for parents with elevated stress levels at baseline.

### Limitations and Future Directions

While results from the present study are promising and merit further research, a few limitations should be considered. Importantly, the current study did not have a control or active comparison group, nor did the current study collect data on additional treatments received by the participants in the follow-up period. Without such data, it can be difficult to determine the extent to which PEERS^®^ for Preschoolers itself was responsible for maintenance of treatment outcomes. Additionally, details on the children’s intellectual or adaptive functioning were not available. Further assessment of such factors in future studies would be fruitful to explore as potential moderators of the magnitude of initial treatment gains or their durability over time following PEERS^®^ for Preschoolers. Diagnostic verification and additional information regarding the participant’s diagnosis, either by comprehensive psychodiagnostic evaluation or chart review, would also strengthen the impact of current findings.

Contrary to previous research on parenting stress in ASD (Hayes & Watson, 2013; Schieve et al., 2007), stress was not clinically elevated in the current sample on the Parental Distress or Parent–Child Dysfunctional Interaction domains. Notably, there was low power and large variation in scores across these domains. Given these limitations, future research with a larger sample size may better elucidate the impact of the PEERS^®^ for Preschoolers program on specific domains in parenting stress. Future studies may also seek to identify whether baseline parenting stress moderates the effect of PEERS^®^ for Preschoolers, as it is possible that those parents with the highest stress may stand to benefit the greatest.

An important caveat to consider when interpreting the results of the present study is that the reference point used to differentiate number of playdates between clinic-referred and community-based samples (i.e., 2.5 playdates per month) was based on samples of elementary school-aged children (Frankel et al., 2010). With the present literature focusing predominantly on behavior during playdates (Koegel et al., 2005; Raulston et al., 2019), we are unaware of research that has quantified a typical number of planned social interactions among community samples of preschool children. However, limited research on play quantity suggests that older children tend to participate in a larger number of informal playdates, as they attain the social communication skills necessary to request and initiate playdates themselves (Ladd & Hart, 1922). Thus, it is possible that 2.5 playdates per month may be an overly stringent criteria for the preschool-age population. The present study would therefore benefit from literature quantifying a typical number of playdates among preschool-age children to assess whether children were already participating in a typical number of social interactions at baseline. Nevertheless, it is promising that children demonstrated improvements in number of playdates per month that sustained to the early elementary years, and at that time, were engaging in playdates at a developmentally normative frequency. This is especially important given that many children with ASD demonstrate pronounced relational challenges by elementary school (Rao et al., 2008).

Parent participation in the intervention may have also influenced parent ratings on outcome measures. Although no significant differences were found between Completers and Non-Completers in demographics or treatment outcomes, it is possible that parents who were more satisfied with the intervention were also more likely to participate in follow-up or report greater improvements from the intervention. Future studies would benefit from rating scales completed by informants who were not involved with the program (e.g., teachers or providers) to remove potential confounds and allow for comparisons between raters. Future efforts may also prioritize collection of direct observational and coding measures to objectively assess treatment outcomes and skills mastery in naturalistic settings.

## Conclusions

Overall, young children with ASD in PEERS^®^ for Preschoolers demonstrated long-term improvements in socialization skills, with areas targeted in the curriculum showing maintenance 1–5 years after program completion. Sustained improvements were likely related to the strategies utilized in the program (e.g., concrete scaffolding of skills, continued practice, and parent involvement) and suggest that young children with ASD are able to learn and maintain important play and friendship-making skills. Considering the persistence of social skills impairments in ASD throughout the lifespan, there is a critical need for social skills interventions in young children that remain beneficial over time. The present study highlights the importance of teaching early play and friendship-making skills to establish a foundation for social relationships in young children with ASD. Additionally, as the field continues to create and test programs for children with ASD, it is necessary that interventions and parent training appropriately meet changing needs across development.

## Data Availability

Coded data for this study is available upon request at the UCLA PEERS Clinic.
